# The predictive value of the vasoactive inotropic score in the postoperative treatment of patients suffering from infective endocarditis

**DOI:** 10.1038/s41598-025-16769-w

**Published:** 2025-10-28

**Authors:** Philipp Schnackenburg, Christopher Ternka, Shekhar Saha, Dietmar Wassilowsky, Christine Kamla, Christoph Mueller, Konstanze Horke, Sven Peterss, Christian Hagl, Dominik Joskowiak

**Affiliations:** 1 Department of Cardiac Surgery, LMU University Hospital, Marchioninistrasse 15, 81377 Munich, Germany; 2 German Centre for Cardiovascular Research (DZHK), Partner Site Munich Heart Alliance, 80802 Munich, Germany; 3 Department of Anesthesiology, LMU University Hospital, Marchioninistrasse 15, 81377 Munich, Germany

**Keywords:** Cardiology, Valvular disease, Bacterial infection

## Abstract

The study aimed to analyze the predictive value of the vasoactive inotropic score (VIS) in the postoperative treatment of patients undergoing valve surgery for infective endocarditis (IE). At our institution, 334 patients underwent valve surgery for IE from 01/2016 to 12/2022. Patients with postoperative MCS device were excluded from this study. Data are presented as medians and 25th – 75th percentiles, or absolute numbers and percentages. Non-survivors had a significantly higher EuroSCORE II preoperatively (4.5 (2.3–9.8)% vs. 12.6 (5.7–20.9)%, *p* < 0.001). In non-survivors, Staphylococcus (89 (32.1%) vs. 20 (54.1%), *p* = 0.01) and Staphylococcus aureus (62 (22.4%) vs. 14(37.8%), *p* = 0.064) were significantly more frequently identified as causative pathogens. Non-survivors had significantly higher IL-6 levels at POD 1, 2 and 4 compared to survivors (*p* < 0.021). Non survivor had significantly higher VIS immediately postoperatively as well as 6 h, 12 h, 24 h, 36 h and 48 h after surgery. In addition, VIS at 48 h was identified as an independent variable associated with non-survival at values > 4.1. When comparing the ROC of lactate, ScvO2 and VIS after 48 h, VIS showed the highest AUC. The VIS is particularly suitable for patients with infective endocarditis due to combined assessment of postoperative cardiovascular dysfunction and IE related inflammatory response. We provided suggestive evidence that the amount of cardiovascular support represented as the VIS has a predictive value regarding mortality in patients undergoing valve replacement for IE. In addition, VIS showed superiority compared to conventional scoring systems for predicting outcome in the intensive care of postoperative IE patients.

## Introduction

Patients suffering from infective endocarditis (IE) are prone to excessive release of cytokines and other inflammatory mediators during surgery as they are exposed to infected material during the removal of vegetations and resection of infected tissue. In addition, surgical trauma, cardiopulmonary bypass, ischemia-reperfusion phenomena, and the effects of general anesthesia can lead to a generalized inflammatory response, vasoplegic reaction and therefore multiorgan failure in patients undergoing cardiac surgery^1,2^. Common ICU scoring systems were not primarily developed for postoperative cardiac surgery patients so that patients’ reactions to the surgery, cardiopulmonary bypass and inflammatory release especially in IE patients are hardly taken into account^3–6^.

The vasoactive-inotropic score (VIS) is calculated from the weighted sum of all administered inotropes and vasoconstrictors and thus reflects the extent of pharmacological support of the cardiovascular system^6,7^. Originally developed for the treatment of septic patients, in recent years it has been shown that a higher VIS value predicts unfavorable outcomes after pediatric cardiac surgery^6–10^. The use of the VIS in postoperative IE patients is supported by its role in assessing the severity of the need for circulatory support, which correlates with morbidity and mortality. While the VIS has traditionally been used as a preoperative indicator, its usefulness in the postoperative setting has been confirmed by studies indicating its prognostic value in critical care, particularly in relation to the intensity of inotropic and vasopressor support, which may indicate the risk of adverse outcomes (e.g. mortality, prolonged ICU stay)^6–8^. In particular, maximum VIS within 24 h of ICU admission (VISmax) has been repeatedly evaluated as a good predictor of adverse outcomes in these patients^6–8^. Although several studies have investigated the predictive value of VIS in pediatric cardiac surgery, there are few studies in adult cardiac surgery patients and no study investigating patients suffering from IE^5,6,8,10,11^.

The aim of the present study was to investigate the predictive value of the VIS in postoperative cardiac surgery patients suffering from IE by a combined assessment of postoperative cardiovascular dysfunction and IE-related inflammatory response.

## Methods

### Study design

All methods were carried out in accordance with relevant guidelines and regulations. This study was approved by the institutional ethics committee of the Ludwig Maximilian University (22–0612). Due to the retrospective nature of the study, the need to obtain the informed consent was waived by the institutional ethics committee of the Ludwig Maximilian University (22–0612). Postoperative treatment and data collection was performed as part of routine patient care. Data acquisition was based on institutional databases and was subsequently anonymized. All data processing described in this study was in accordance with the institutional ethics board and national data safety regulations. Between January 2016 and December 2022, 334 consecutive patients underwent valve surgery for IE at our institution. Patients with postoperative mechanical circulatory support (MCS) devices were excluded from this study.

### Scoring systems and definitions

The European System for Cardiac Operative Risk Evaluation II (EuroSCORE II) was used to predict the risk of perioperative mortality^12^. VIS was calculated according to the following formula: [Dopamine dose (µg/kg/min)] + [Dobutamine dose (µg/kg/min)] + [50*Levosimendan dose (µg/kg/min)] + [100*Noradrenaline dose (µg/kg/min)] + [100*Adrenaline dose (µg/kg/min)] + [10000*Vasopressin dose (U/kg/min)] + [10*Milrinone dose (µg/kg/min)]^13^. Cardiac output was calculated according to modified Fick formula: CO (l/min) = [VO_2_] / [*13*,4*Hb*(SaO_*2*_*-ScvO*_*2*_*)*]. Cardiac index was calculated by the following formula: CI(l/min/m^2^) = CO / BSA^14–16^.

### Statistical analysis

Data were analyzed using the IBM SPSS Statistics Data Editor^®^ version 29 (IBM Corp. Released 2022. IBM SPSS Statistics, Version 29.0. Armonk, NY: IBM Corp.). Data were tested for normal distribution using the Kolmogorov-Smirnov test with Lillefors correction. Categorical variables were analyzed using the Chi-Squared and Fisher‘s exact test and continuous variables using the Mann-Whitney-U test. Multivariable analysis included logistic regression using a forward stepwise (conditional) model, where significance for entry was set at *p* < 0.05 and significance for exit was *p* < 0.10. The regression model was verified using the goodness of fit test as well as tests for autocorrelation, multicollinearity, and heteroscedasticity. Illustrations were prepared using GraphPad Prism (GraphPad Software Inc., San Diego, CA, USA). Data are presented as medians (25–75th quartiles) or as absolute numbers (percentages) unless otherwise specified.

## Results

### Patient characteristics, comorbidities and preoperative condition

Patient characteristics and comorbidities are listed in Table 1. There were no differences in age and gender distribution between survivors and non-survivors. Non-survivors had a significantly higher EuroSCORE II preoperatively (4.5 (2.3–9.8) vs. 12.6% (5.7–20.9), *p* < 0.001). Non-survivors were significantly more likely to have IDDM (61 (20.6%) vs. 18 (45.0%), *p* < 0.001), PAD (17 (5.8%) vs. 7 (17.5%), *p* = 0.015) and CKD (74 (25.0%) vs. 22 (55.0%), *p* < 0.001). Non-survivors were significantly more frequently ventilated preoperatively (13 (4.4%) vs. 7 (17.5%), *p* = 0.005).

### IE specific data

IE-specific data and the preoperative status are listed in Table 1. There was no statistically significant difference between the cohorts in terms of the affected heart valves. In non-survivors, Staphylococcus sp. (89 (32.1%) vs. 20 (54.1%), *p* = 0.01) and Staphylococcus aureus (62 (22.4%) vs. 14 (37.8%), *p* = 0.064) were significantly more frequently identified as causative pathogens. Conversely, survivors suffered statistically significant more frequently from streptococcal endocarditis (95 (34.4%) vs. 6 (16.2%), *p* = 0.038).

**Table 1:** Patient characteristics comorbidities and details of infective endocarditis.

	Survivors (*n* = 294)	Non-survivors (*n* = 40)	*p*-value
Age (years)	67 (58–73)	67 (50–79)	0.173
Male (%)	240 (81.1)	29 (72.5)	0.209
BMI (kg/m^2^)	24 (22–28)	25 (23–32)	0.359
EuroSCORE II (%)	4.5 (2.3–9.8)	12.6 (5.7–20.9)	**< 0.001**
Smoking (%)	84 (28.5)	12 (30.0)	0.853
Alcohol abuse (%)	28 (9.5)	6 (15.0)	0.270
Intravenous drug abuse (%)	12 (4.1)	0 (0.0)	0.373
Co-morbidities (%)			
Preoperative stroke	66 (22.3)	12 (30.0)	0.318
Arterial hypertension	205 (69.3	27 (67.5)	0.856
IDDM	61 (20.6)	18 (45.0)	**0.001**
Dyslipidemia	113 (38.3)	19 (47.5)	0.302
Coronary artery disease	95 (32.3)	19 (47.5)	0.220
Recent PCI/PTCA	27 (9.1)	5 (12.5)	0.563
Atrial fibrillation	83 (28.4)	12 (30.0)	0.853
S/p pacemaker	38 (12.8)	7 (17.5)	0.456
COPD	32 (10.9)	8 (20.0)	0.116
PAD	17 (5.8)	7 (17.5)	**0.015**
Chronic kidney disease	74 (25.0)	22 (55.0)	**< 0.001**
Dialysis	17 (5.8)	2 (5.0)	1.000
Malignancy	49 (17.5)	10 (26.3)	0.188
Preoperative medication (%)			
Steroid treatment	5 (1.8)	2 (5.3)	0.199
Vitamin K antagonists	22 (8.2)	4 (12.1)	0.506
NOAC	43 (16.0)	9 (27.3)	0.1139
SAPT	77 (28.6)	12 (36.4)	0.418
DAPT	12 (4.1)	1 (3.0)	0.899
Previous open cardiac surgery (%)	98 (33.4)	17 (42.5)	0.289
Previous TAVR (%)	18 (6.2)	2 (5.3)	1.000
Previous endocarditis (%)	17 (10.7)	1 (6.7)	1.000
Preoperative ventilation (%)	13 (4.4)	7 (17.5)	**0.005**
Affected valves (%)			
Aortic valve	183 (61.8)	28 (70)	0.385
Mitral valve	119 (40.2)	17 (42.5)	0.864
Tricuspid valve	14 (4.7)	1 (2.5)	1.000
Pulmonary valve	0 (0.0)	1 (2.5)	0.119
Prosthetic valve	100 (33.9)	17 (42.5)	0.293
Pathogens (%)			
BCNIE	30 (10.8)	4 (10.8)	1.000
Staphylococcus sp.	89 (32.1)	20 (54.1)	**0.010**
Staphylococcus aureus	62 (22.4)	14 (37.8)	0.064
Enterococcus sp.	39 (14.1)	6 (16.2)	0.802
Streptococcus sp.	95 (34.3)	6 (16.2)	**0.038**
Other Gram positive	22 (7.9)	2 (5.4)	0.752
Gram negative	10 (3.6)	1 (2.7)	1.000
Candida sp.	3 (1.1)	1 (2.7)	0.397

Data are presented as medians (25–75th quartiles) or as absolute numbers (percentages): NYHA: New York Heart association; IDDM: insulin dependent diabetes mellitus; PTCA: percutaneous transluminal coronary angioplasty; PCI: percutaneous coronary intervention; COPD: chronic obstructive pulmonary disease; PAD: peripheral arterial disease; NOAC: novel oral anticoagulants; SAPT: single antiplatelet therapy; DAPT: dual antiplatelet therapy; TAVR: trans-aortic valve replacement; BCNIE: blood culture negative infective endocarditis; Marked p-values are two-sided significant at least at the 0.05 level.

### Pre- and postoperative laboratory results

Detailed pre- and postoperative laboratory results are listed in Table 2. Non-survivors had statistically significant higher bilirubin levels (mg/dl) preoperative and on POD 0 to 4, statistically significant higher thrombocyte levels (G/l) on POD 0, 1 and 4, statistically significant higher leukocyte levels (G/l) preoperative and on POD 2, 3 and 4, statistically significant higher IL-6 levels (pg/ml) preoperative and on POD 1, 2 and 4 and statistically significant higher C-reactive protein (mg/dl) preoperative compared to survivors.

**Table 2:** Laboratory results.

	Survivors (*n* = 294)	Non-survivors (*n* = 40)	*p*-value
Preoperative bilirubin (mg/dl)	0.5 (0.4–0.8)	0.7 (0.4-1.0)	**0.046**
Postoperative bilirubin (mg/dl)	1.1 (0.8–1.6)	1.6 (1.3–2.4)	**< 0.001**
Bilirubin POD1 (mg/dl)	1.3 (0.9–2.1)	3.7 (3.3–4.2)	**0.002**
Bilirubin POD2 (mg/dl)	0.9 (0.5–1.6)	2.4 (1.0–4.0)	**< 0.001**
Bilirubin POD3 (mg/dl)	0.6 (0.3-1.0)	1.7 (1-3.4)	**< 0.001**
Bilirubin POD4 (mg/dl)	0.5 (0.4–0.8)	1.5 (0.7–3.9)	**< 0.001**
Preoperative leucocytes (G/l)	8.5 (6.2–11)	10 (7.9–11.9)	**0.013**
Postoperative leucocytes (G/l)	20.4 (14.8–25.3)	21 (14.6–28.6)	0.794
Leucocytes POD1 (G/l)	14.7 (11.2–20.1)	17.8 (12.7–23.2)	0.057
Leucocytes POD2 (G/l)	11.5 (9.0-14.6)	13.8 (10.9–19.6)	**0.010**
Leucocytes POD3 (G/l)	12.0 (9.4–15.8)	16.5 (10.8–20.1)	**< 0.001**
Leucocytes POD4 (G/l)	10.8 (7.8–14.4)	15.5 (10.0-21.1)	**< 0.001**
Preoperative thrombocytes (G/l)	253 (183–319)	228 (149–289)	0.120
Postoperative thrombocytes (G/l)	193 (151–249)	169 (114–223)	**0.025**
Thrombocytes POD1 (G/l)	186 (137–234)	154 (117–203)	**0.028**
Thrombocytes POD2 (G/l)	173 (131–219)	157 (107–193)	0.112
Thrombocytes POD3 (G/l)	157 (121–210)	147 (108–200)	0.157
Thrombocytes POD4 (G/l)	160 (121–212)	132 (100–179)	**0.020**
Preoperative interleukin-6 (pg/ml)	26 (13–47)	42 (25–108)	**0.045**
Postoperative interleukin-6 (pg/ml)	185(89–267)	221 (118–259)	0.450
Interleukin-6 POD1 (pg/ml)	98 (61–167)	209 (108–328)	**< 0.001**
Interleukin-6 POD2 (pg/ml)	98 (65–162)	144 (112–269)	**< 0.001**
Interleukin-6 POD3 (pg/ml)	65 (37–109)	83 (45–150)	0.136
Interleukin-6 POD4 (pg/ml)	44 (29–71)	66 (42–133)	**0.021**
Preoperative C-reactive protein (mg/dl)	4.1 (1.7–9.5)	5.9 (3.6–11.6)	**0.017**
Postoperative C-reactive protein (mg/dl)	2.5 (1.1–5.1)	2.4 (1.6–1.8)	0.773
C-reactive protein POD1 (mg/dl)	4.9 (2.3–8.3)	4.4 (3.1–6.5)	0.809
C-reactive protein POD2 (mg/dl)	6.9 (4.6–10)	5.8 (3.7–9.9)	0.537
C-reactive protein POD3 (mg/dl)	12.8 (9.2–16.9)	11.5 (9.6–19.3)	0.754
C-reactive protein POD4 (mg/dl)	12.7 (8.1–16.9)	11.5 (7.9–17.8)	0.715

Data are presented as medians (25–75th quartiles) or as absolute numbers (percentages): POD: post-operative day; Marked p-values are two-sided significant at least at the 0.05 level.

### Postoperative morbidities

Postoperative morbidities are listed in Table 3. Non-survivors had a significantly longer duration of ventilation (19 (13–25) vs. 44 h (19–100), *p* < 0.001), had a significantly higher rate of cerebrovascular events (50 (17.1%) vs. 13 (35.1%), *p* = 0.014) and were significantly more likely to require dialysis (28 (9.5%) vs. 11 (29.7%), *p* < 0.001). Non-survivors were significantly more likely to require red blood cell concentrates (3 (1–4) vs. 4 units (2–7), *p* < 0.001), platelet concentrates (0 (0–1) vs. 3 units (1–4), *p* < 0.001) and fresh frozen plasma (0 (0–4) vs. 12 units (7–18), *p* < 0.001).

**Table 3:** Postoperative morbidities.

	Survivors (*n* = 294)	Non-survivors (*n* = 40)	*p*-value
Duration of ventilation (hours)	19 (13–25)	44 (19–100)	**< 0.001**
ICU stay (days)	5 (3–7)	6 (3–10)	0.062
Pneumonia (%)	44 (25.1)	8 (20.0)	0.082
Tracheostomy (%)	17 (5.8)	2 (5.4)	1.000
Renal replacement therapy (%)	28 (9.5)	11 (29.7)	**0.001**
Surgical site infection (%)	4 (0.0)	0 (0.0)	1.000
Adverse cerebrovascular events (%)	50 (17.1)	13 (35.1)	**0.014**
Permanent pacemaker implantation (%)	42 (14.3)	1 (2.7)	0.065
PRBCs transfused (units)	3 (1–4)	4 (2–7)	**< 0.001**
Platelets transfused (units)	0 (0–1)	3 (1–4)	**< 0.001**
FFPs transfused (units)	0 (0–4)	12 (7–18)	**< 0.001**

Data are presented as medians (25–75th quartiles) or as absolute numbers (percentages): ICU: intensive care unit. PRBC: packed red blood cells; FFP: fresh frozen plasma; Marked p-values are two-sided significant at least at the 0.05 level.

### Vasoactive inotropic score and postoperative vasopressor therapy

**Fig. 1:**
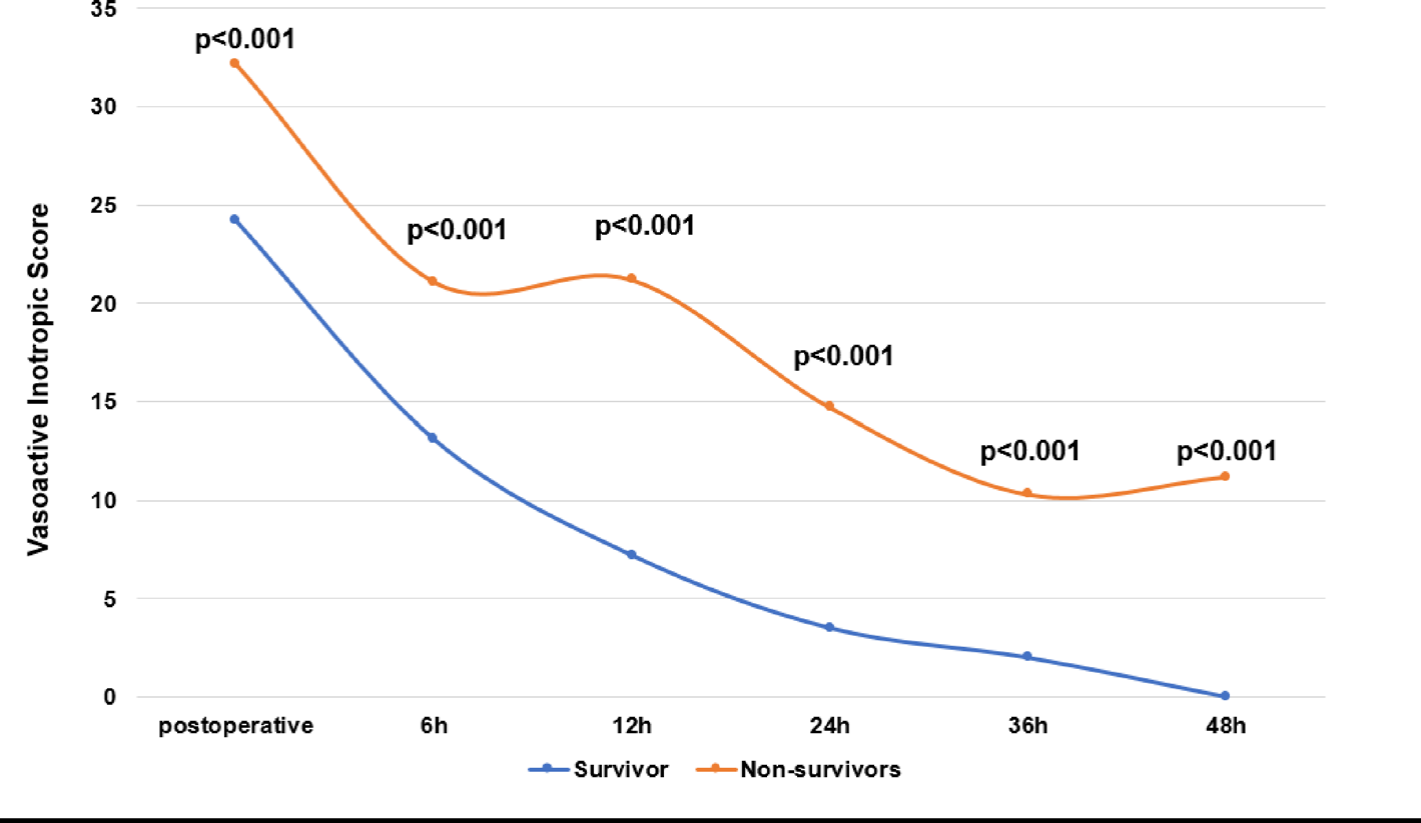
Vasoactive inotropic score. Vasoactive inotropic score was calculated postoperative (= admission on ICU), 6 h, 12 h, 24 h, 36 h, and 48 h after surgery. Data are presented as medians (25–75th quartiles) or as absolute numbers (percentages). Marked p-values are two-sided significant at least at the 0.05 level.

**Fig. 2:**
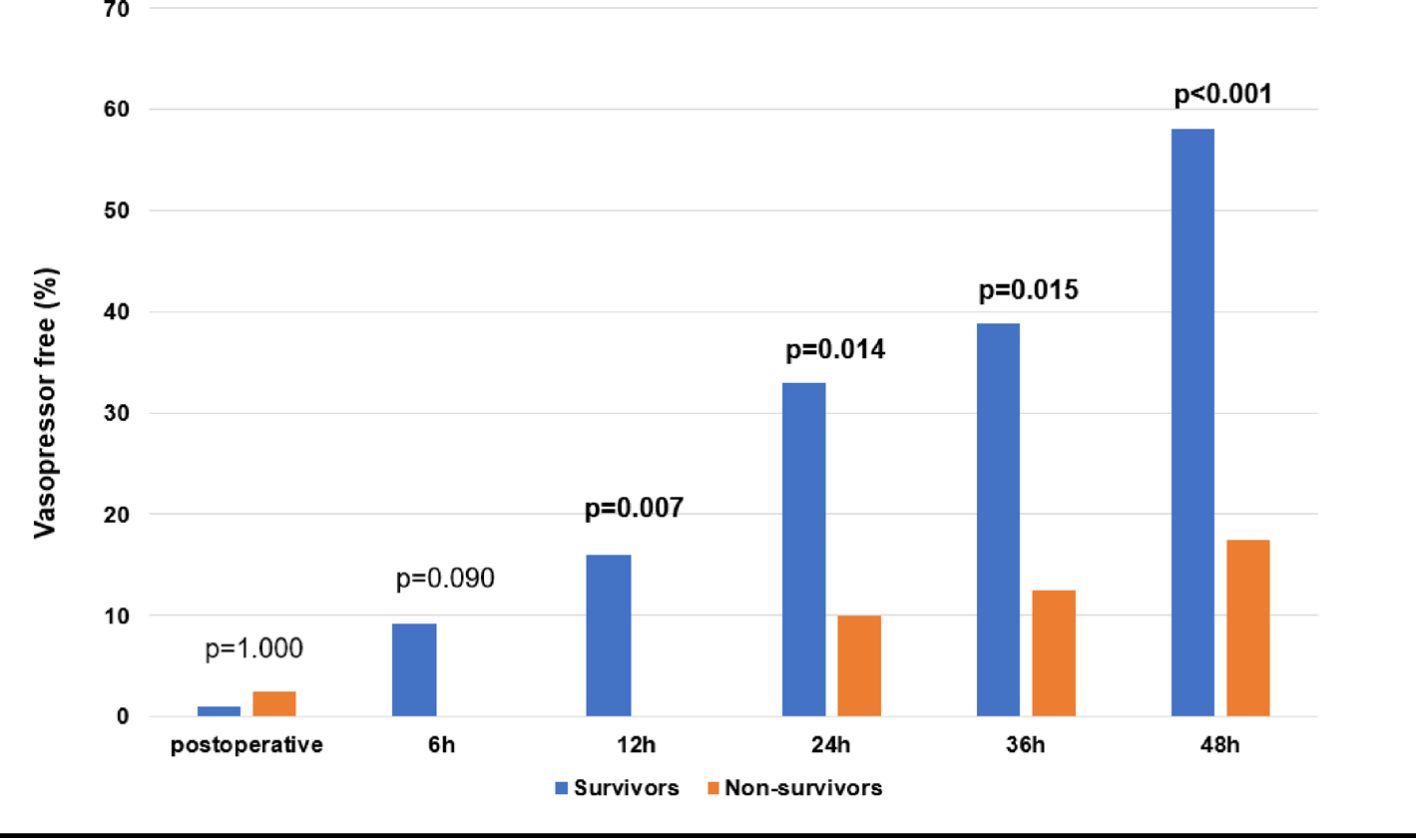
Freedom from vasopressors. Survivors and Non-survivors compared in terms of freedom from vasopressors postoperative (= admission on ICU), 6 h, 12 h, 24 h, 36 h, and 48 h after surgery. Data are presented as medians (25–75th quartiles) or as absolute numbers (percentages). Marked p-values are two-sided significant at least at the 0.05 level.

The VIS course in the first 48 h after surgery is shown in Fig. 1; Table 4. Non-survivors had a statistically significant higher vasoactive inotropic score compared to survivors immediately postoperatively as well as 6 h, 12 h, 24 h, 36 h, and 48 h after surgery. Postoperative freedom from vasopressor therapy in the first 48 h after surgery is shown in Fig. 2; Table 4. Survivors were significantly more likely to be free from vasopressors at 6 h, 12 h, 24 h, 36 h, and 48 h compared to non-survivors.

### Hemodynamic course

**Fig. 3:**
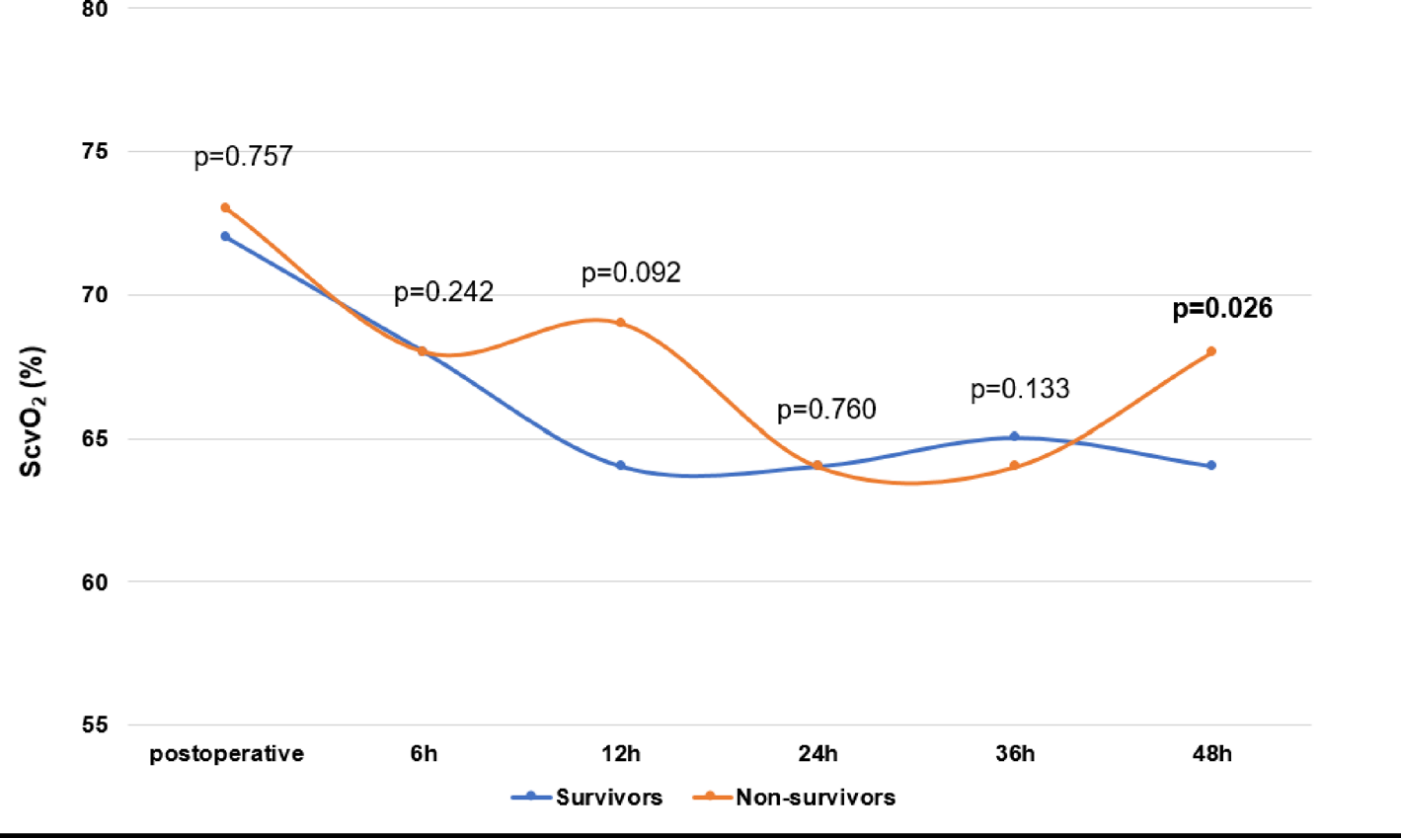
Central venous saturation. Central venous saturation (ScvO_2_) was measured postoperative (= admission on ICU), 6 h, 12 h, 24 h, 36 h, and 48 h after surgery. Data are presented as medians or as absolute numbers (percentages). Marked p-values are two-sided significant at least at the 0.05 level.

**Fig. 4:**
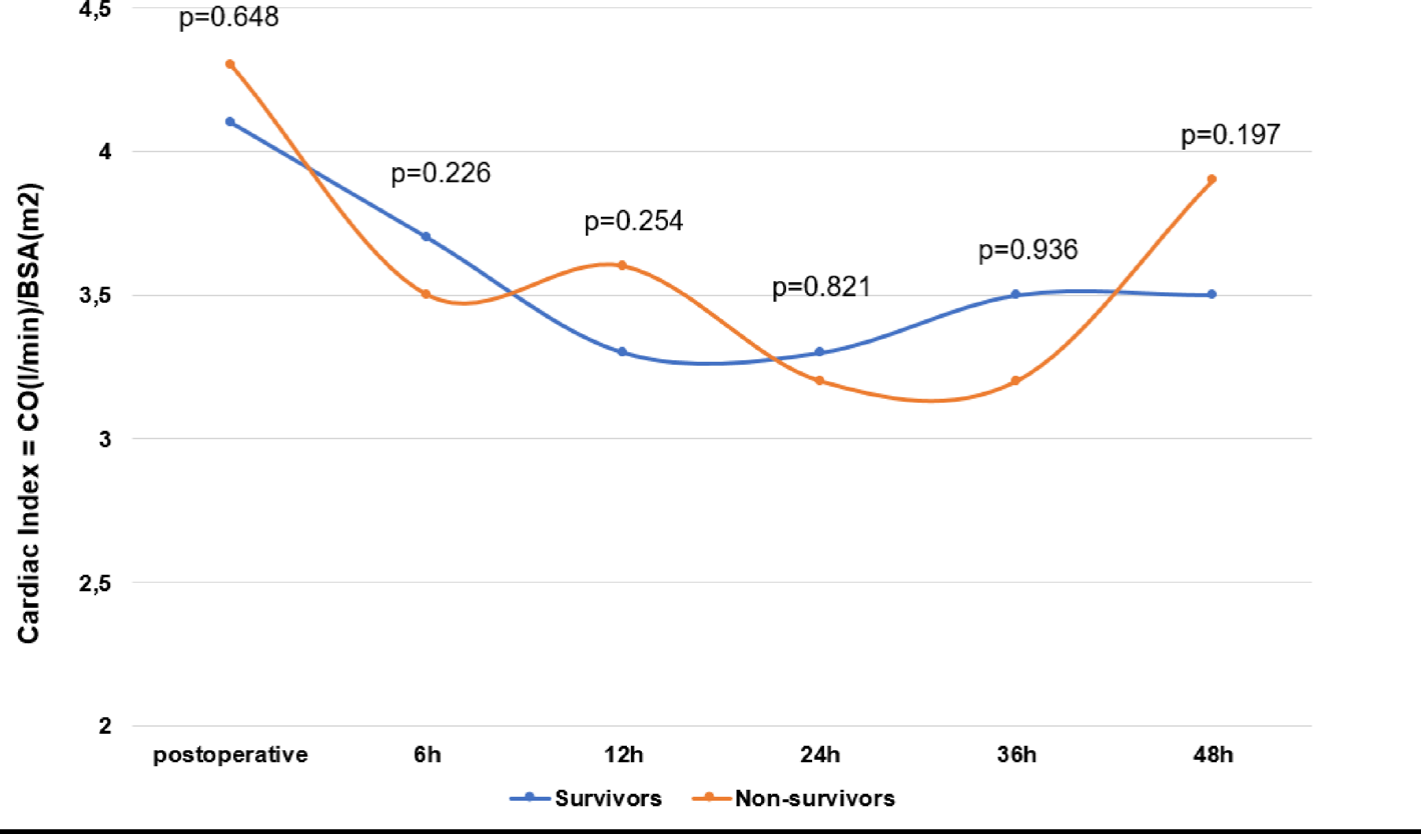
Cardiac Index. Cardiac index was calculated postoperative (= admission on ICU), 6 h, 12 h, 24 h, 36 h, and 48 h after surgery. Data are presented as medians or as absolute numbers (percentages). Marked p-values are two-sided significant at least at the 0.05 level.

The hemodynamic course including cardiac output, cardiac index and ScvO_2_ (%) in the first 48 h after surgery is shown in Figs. 3 and 4; Table 4. There was no significant difference in cardiac output and cardiac index between survivors and non-survivors. At 48 h after surgery, non-survivors had a significantly higher ScvO_2_ (64 (59–70) % vs. 68 (60–73) %, *p* = 0.026).

### Lactate

**Fig. 5:**
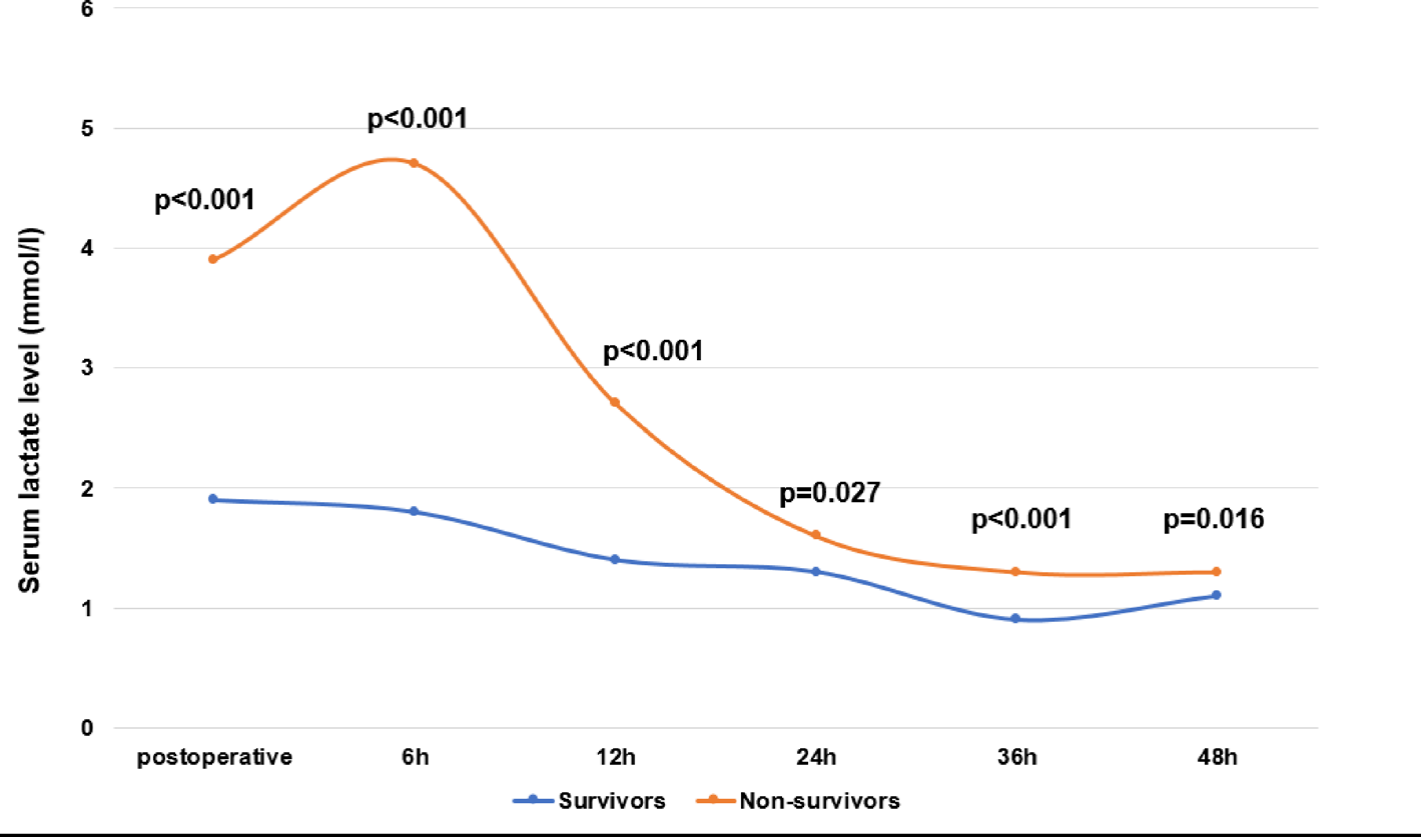
Serum lactate. Serum lactate levels were measured postoperative (= admission on ICU), 6 h, 12 h, 24 h, 36 h, and 48 h after surgery. Data are presented as medians or as absolute numbers (percentages). Marked p-values are two-sided significant at least at the 0.05 level.

The lactate levels in the first 48 h after surgery are shown in Fig. 5; Table 4. Non-survivors had significantly higher lactate levels (mmol/l) compared to survivors immediately postoperatively and 6 h, 12 h, 24 h, 36 h and 48 h after surgery.

**Table 4:** Vasoactive inotropic score and hemodynamic course.

	Survivors (*n* = 294)	Non-survivors (*n* = 40)	*p*-value
Postoperative VIS	24.2 (13.5–32.0)	32.0 (20.7–50.1)	**< 0.001**
6 h VIS	13.1 (4.2–19.7)	21.1 (14.7–32.0)	**< 0.001**
12 h VIS	7.2 (2.2–14.1)	21.2 (10.2–29.4)	**< 0.001**
24 h VIS	3.5 (0.0-8.9)	14.7 (6.9–24.7)	**< 0.001**
36 h VIS	2.0 (0.0-7.6)	10.3 (3.6–19.7)	**< 0.001**
48 h VIS	0.0 (0.0-4.2)	11.2 (2.7–14.0)	**< 0.001**
Vasopressor free postoperative (%)	3 (1.0)	1 (2.5)	1.000
Vasopressor free 6 h (%)	27 (9.2)	0 (0.0)	0.090
Vasopressor free 12 h (%)	47 (16.0)	0 (0.0)	**0.007**
Vasopressor free 24 h (%)	97 (33.0)	4 (10.0)	**0.014**
Vasopressor free 36 h (%)	114 (38.8)	5 (12.5)	**0.015**
Vasopressor free 48 h (%)	171 (58.1)	7 (17.5)	**< 0.001**
Postoperative cardiac output (l/min)	8.0 (6.2–10.4)	8.2 (6.1–9.8)	0.599
6 h cardiac output (l/min)	7.2 (5.9–9.2)	6.9 (6.1–9.5)	0.278
12 h cardiac output (l/min)	6.4 (5.4-8.0)	6.6 (5.3–10.2)	0.475
24 h cardiac output (l/min)	6.6 (5.7–7.9)	6.7 (4.9–8.1)	0.594
36 h cardiac output (l/min)	7.0 (6.0-8.6)	5.6 (6.5–8.3)	0.750
48 h cardiac output (l/min)	7.0 (5.6–8.2)	6.9 (6.0-8.2)	0.658
Postoperative cardiac index (l/min/m^2^)	4.1 (3.2–5.2)	4.3 (2.9–5.3)	0.648
6 h cardiac index (l/min/m^2^)	3.7 (3.1–4.9)	3.5 (3.1–5.2)	0.226
12 h cardiac index (l/min/m^2^)	3.3 (2.8–4.1)	3.6 (2.8–5.3)	0.254
24 h cardiac index (l/min/m^2^)	3.4 (2.9–4.1)	3.2 (2.6–4.8)	0.821
36 h cardiac index (l/min/m^2^)	3.5 (3.0-4.3)	3.2 (2.8-5.0)	0.936
48 h cardiac index (l/min/m^2^)	3.5 (2.9–4.1)	3.9 (2.9–4.6)	0.197
Postoperative ScvO_2_ (%)	72 (67–77)	73 (66–78)	0.757
6 h ScvO_2_ (%)	68 (63–74)	68 (64–77)	0.242
12 h ScvO_2_ (%)	64 (59–70)	69 (61–75)	0.092
24 h ScvO_2_ (%)	64 (60–69)	64 (58–72)	0.760
36 h ScvO_2_ (%)	65 (59–70)	64 (58–75)	0.133
48 h ScvO_2_ (%)	64 (59–70)	68 (60–73)	**0.026**
Postoperative lactate level (mmol/l)	1.9 (1.3-3.0)	3.9 (2.2–4.9)	**< 0.001**
6 h lactate level (mmol/l)	1.8 (1.2–3.7)	4.7 (2.6–6.2)	**< 0.001**
12 h lactate level (mmol/l)	1.4 (1.0-2.6)	2.7 (1.9–5.9)	**< 0.001**
24 h lactate level (mmol/l)	1.3 (1.1–1.9)	1.6 (1.2–2.8)	**0.027**
36 h lactate level (mmol/l)	0.9 (0.8–1.2)	1.3 (1.0-1.8)	**< 0.001**
48 h lactate level (mmol/l)	1.1 (0.9–1.5)	1.3 (1.1–2.2)	**0.016**

Data are presented as medians (25–75th quartiles) or as absolute numbers (percentages). VIS: vasoactive inotropic score. ScvO_2_: central venous saturation; Marked p-values are two-sided significant at least at the 0.05 level.

### ROC analysis

**Fig. 6:**
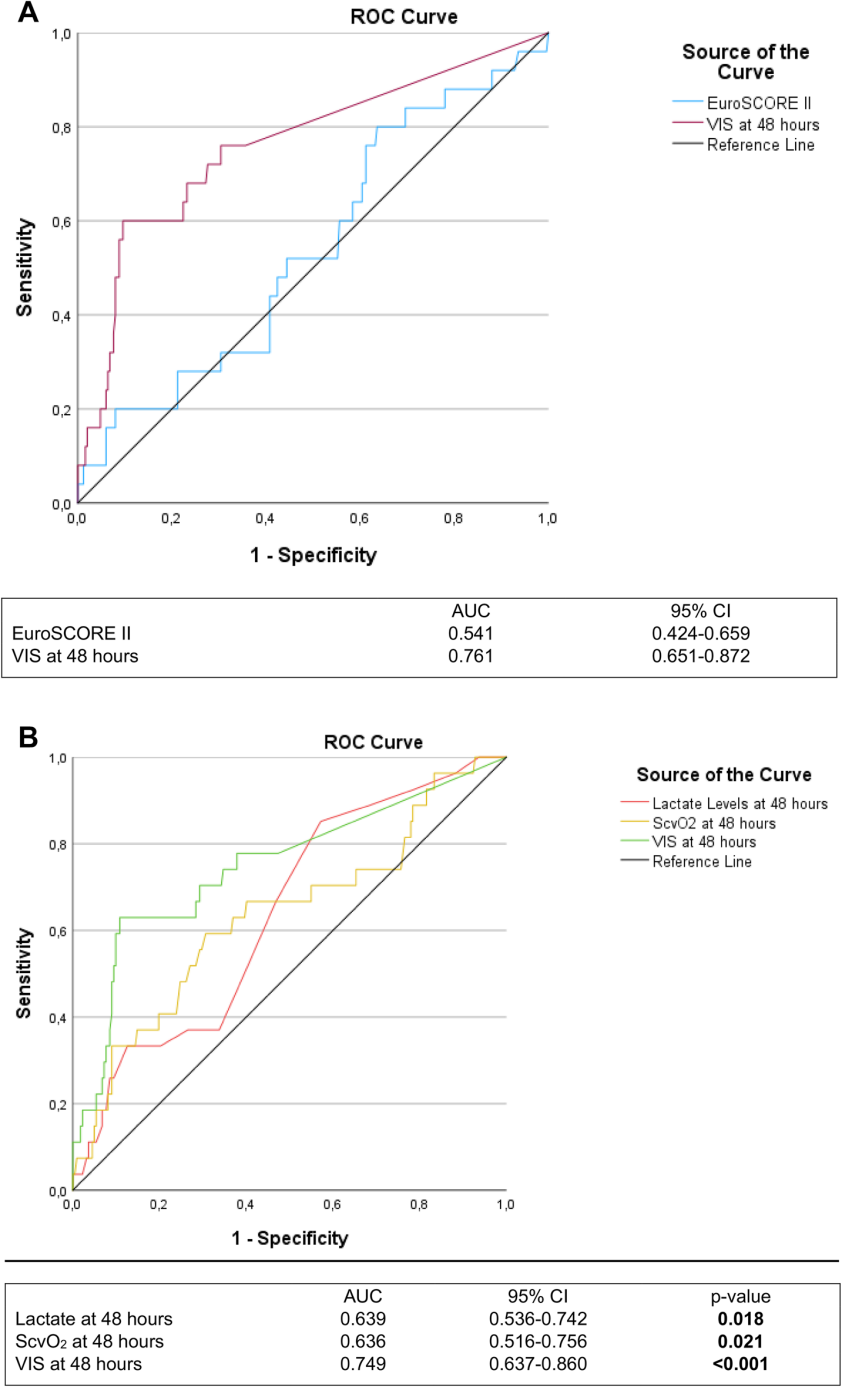
(**A**) ROC sensitivity analysis. Receiver operating characteristic analysis. AUC: Area under the curve; CI: Confidence interval; VIS: Vasoactive inotropic score. (**B**) ROC analysis. Receiver operating characteristic analysis. AUC: Area under the curve; CI: Confidence interval; ScvO_2_: Central venous saturation; VIS: Vasoactive inotropic score; Marked p-values are two-sided significant at least at the 0.05 level.

ROC analysis is shown in Fig. 6A and B. In a sensitivity analysis the ability of EuroSCORE II for predicting non-survival was poor (AUC 0.541; 95% CI 0.424–0.658) (Fig. 6A). VIS after 48 h (AUC: 0.749, 95%CI: 0.637–0.860, *p* < 0.001); ScvO_2_ after 48 h (AUC: 0.636, 95%CI: 0.516–0.756, *p* = 0.021) and lactate after 48 h (AUC: 0.639, 95%CI: 0.536–0.742, *p* = 0.018) independently predicted non-survival. VIS at 48 h had the highest AUC (Fig. 6B).

### Multivariate models

**Fig. 7:**
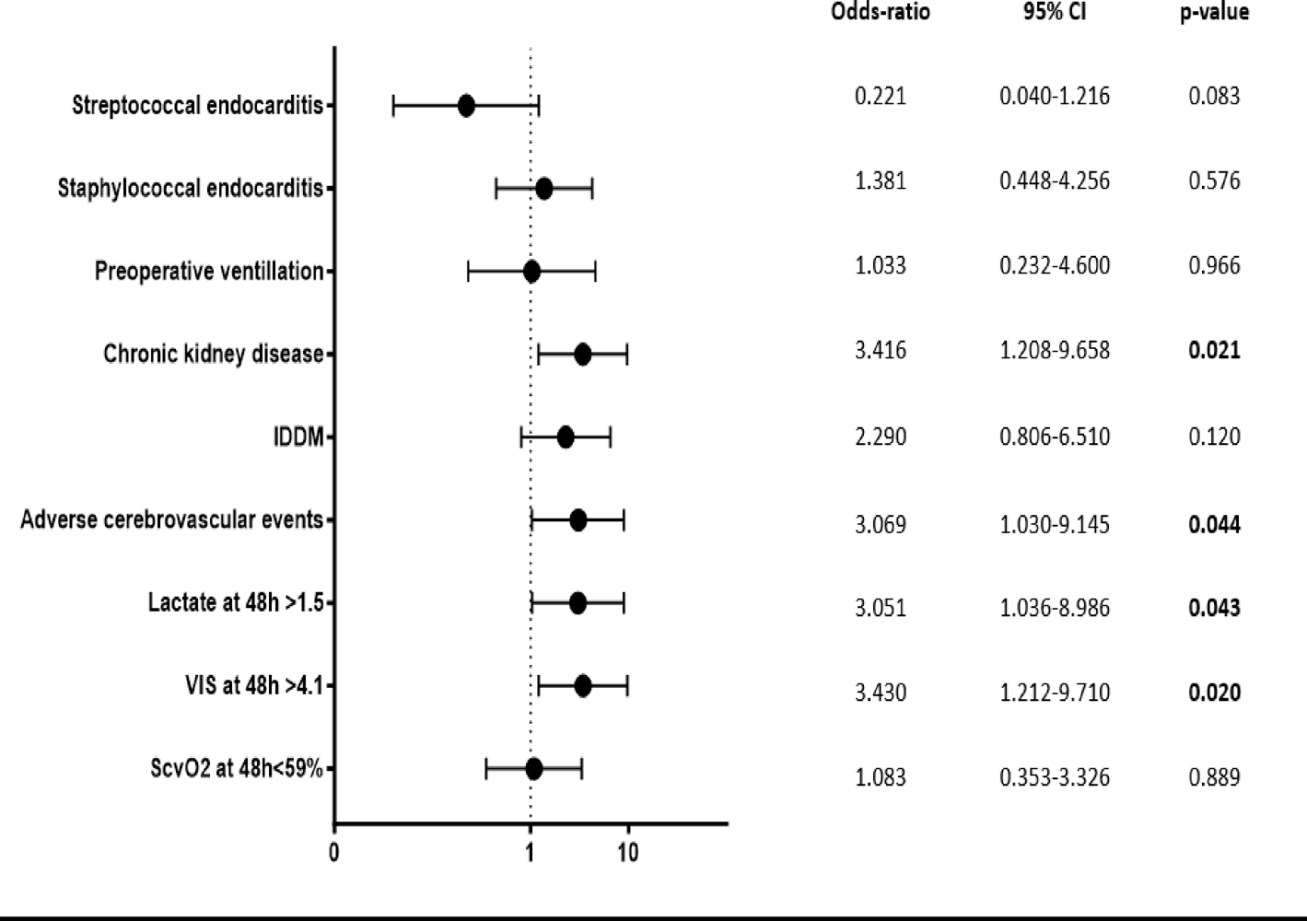
Prediction of mortality. Multivariate analysis: CI: Confidence interval; CKD: Chronic kidney disease; IDDM: Insulin dependent diabetes mellitus; VIS: Vasoactive inotropic score; ScvO_2_: Central venous saturation; Marked p-values are two-sided significant at least at the 0.05 level.

The variables that were independently associated with non-survival in the multivariate analysis are shown in Fig. 7. CKD (OR: 3.416, 95% CI: 1.208–9.658, *p* = 0.021), lactate at 48 h > 1.5 (OR: 3.051, 95% CI: 1.036–8.986, *p* = 0.043) and VIS at 48 h > 4.1 (OR: 3.430, 95% CI: 1.212–9.710, *p* = 0.020) were independently associated with non-survival.

## Discussion

This is the first study to assess the predictive value of VIS calculated in the postoperative care of patients suffering from infective endocarditis. The VIS, primarily used in the intensive care treatment of septic patients, has been shown to be a good predictor for poor clinical outcome after cardiac surgery in various patient groups^5–8,11,17^. Studies repeatedly described, that an increased requirement for vasoactive and inotropic agents during the first 48 h after cardiac surgery can successfully predict the risk of postoperative complications, including mortality up to one year^5,6,11,17^.

The principal findings of the present study are as followed: (1) The VIS at 48 h independently predicted mortality after cardiac surgery in IE patients and (2), the VIS is particularly suitable for patients with infective endocarditis due to combined assessment of postoperative cardiovascular dysfunction and IE related inflammatory response.

The present study showed that an increased need for vasoactive and inotropic agents, quantified by the VIS, was significantly associated with non-survival after surgical treatment of IE. The VIS was significantly increased in patients expiring during hospital stay at almost any time within the first 48 h and a score above 4.1 was identified as independent threshold of mortality. When comparing lactate levels, SvO_2_, and VIS after 48 h, VIS showed the highest predictive value in terms of mortality.

Surgical trauma, cardiopulmonary bypass, ischemia-reperfusion phenomena, and the effects of general anesthesia cause a vasoplegic reaction that can lead to a generalized inflammatory response and multiorgan failure^1,2^. Acute cardiovascular dysfunction is anticipated in 20% or even more patients in the perioperative period of cardiac surgery^18^. At the same time variable degrees of left ventricular dysfunction have been observed in 25–50% of septic shock patients^18^. The combination of all these effects makes the postoperative treatment of IE patients challenging. IE patients are prone to excessive release of cytokines and other inflammatory mediators during surgery as they are exposed to infected material during the removal of vegetations and resection of infected tissue. There is ample evidence that the following increase in cytokine levels correlates with the severity of postoperative organ dysfunction in IE patients^19–21^. In sepsis, high levels of proinflammatory cytokines (i.e., IL-6, IL-8, IL-18, and IL-1β) have been associated with higher mortality, a similar correlation in IE patients can be assumed^19–21^. The present study confirms this correlation. There was a significant association between preoperative as well as POD 1, POD 2 and POD 4 elevated IL6 levels and non-survivors. The combination of cardiovascular dysfunction as well as CPB- and IE-associated inflammation in the postoperative assessment of IE patients is not yet addressed by conventional ICU scoring systems. The analysis of vasoactive and inotropic support using the VIS, which reflects both cardiac dysfunction and inflammation-related vasoplegia, can close this gap.

Patients with critical cardiovascular dysfunction often require a combination of vasoactive and inotropic agents. However, the underlying cause of this dysfunction can vary, particularly in postoperative IE patients. As previously described, the condition may also has an inflammatory or surgical etiology in addition to purely cardiovascular causes. The VIS reflects the extent of hemodynamic support required, with a negative correlation between VIS and clinical outcomes^13^. In the present study, there was no significant correlation between non-survival and a median cardiac index of > 3 l/min/m^2^ for all measurements, so therapeutic success in terms of guideline directed postoperative hemodynamic management (MAP > 65mmHg; CI > 2.1 l/min/m^2^) can be assumed but did not prevent non-survivors from having poor outcome^22^. Other studies already confirmed that myocardial function is depressed in sepsis despite hemodynamic measurements showing increased cardiac output^18^. Furthermore, not only macro- but also microcirculatory dysfunction may contribute to poor outcome in septic patients. Even if increased CO initially compensates for vasodilation and fluid shifts, it does not ensure adequate tissue perfusion. Microcirculatory disorders, such as endothelial dysfunction, leukocyte adhesion and microthrombosis, impair blood flow at the capillary level and prevent adequate tissue oxygenation^23,24^.

Several mechanisms may explain the increased mortality in postoperative patients caused by a high amount of vasoactive support in a vicious circle. This includes increased myocardial oxygen consumption induced by inotropic support and an increased risk for cardiac arrhythmias^18,25^. In addition, low cardiac output may be a result of cardiac tamponade, so inotropes may mask this by initially improving hemodynamics and therefore delaying early surgical intervention^25^. In addition, catecholamines are associated with reduced metabolic efficiency as they promote the oxidation of fatty acids over glucose^25^. This may be a further barrier to optimal cardiac performance. Studies have also found an association with increased bacterial growth, increased bacterial virulence, biofilm formation, insulin resistance and hyperglycemia^25,26^. If this vicious circle is not broken within the first 48 h, the outcome will be poor.

VIS represents the extent of medical inotropic and vasoactive support as a therapeutic necessity in the complex treatment of postoperative and IE-associated circulatory insufficiency as well as the side effects caused by this amount of support, which explains its particular suitability for this special patient group.

Compared to postoperative lactate levels and ScvO_2_, which are commonly used in postoperative monitoring of cardiac surgery patients, the VIS showed superiority in predicting mortality after 48 h. In the present study, lactate, which can be elevated postoperatively due to a transient decrease in systemic organ perfusion, e.g. due to high vasopressor demand, consistently showed a significant correlation with mortality, albeit with a pronounced range of variation and is considered a comparatively unspecific outcome parameter^11,27,28^. ScvO2 levels only showed a significant correlation with non-survival after 48 h and surprisingly they were significantly increased compared to survivors. Studies have shown that ScvO2 levels can be elevated in septic patients due to microcirculatory dysfunction and insufficient tissue oxygen consumption^29,30^. This confirms, that ScvO2 levels especially in septic patients may not always be a reliable indicator for tissue perfusion or outcome and a comprehensive assessment of macro- and microcirculatory parameters is warranted^29,30^. In addition, ScvO_2_ levels are considered susceptible to disturbances caused by circulatory stress and analgosedation, which preferentially redistribute the blood towards the heart muscle and brain^31^.

EuroSCORE II was significantly associated with non-survival in the univariate analysis. However, evaluating its predictive performance in the ROC analysis revealed only limited discriminatory ability, especially when compared to VIS at 48 h. We therefore decided to include key components of EuroSCORE II such as CKD, IDDM and preoperative mechanical ventilation as individual variables. Including both the composite score and its components would have introduced substantial collinearity and impaired model stability. These findings suggest that the VIS provides superior predictive accuracy in this clinical context.

Unlike traditional outcome-based parameters which are more general and consider different organ systems, the VIS focuses specifically on the severity of circulatory support. The VIS captures changes in circulatory support in real time, may providing an early and specific indicator of adverse outcomes such as mortality, prolonged ICU stay or the need for additional interventions. Further studies comparing the VIS with other outcome markers such as SOFA or SAPS II could provide additional insight into its role as a complementary tool in predicting postoperative outcomes.

## Conclusion

The VIS is particularly suitable for patients with infective endocarditis due to combined assessment of postoperative cardiovascular dysfunction and IE related inflammatory response. We provided suggestive evidence that the amount of cardiovascular support represented by the VIS has a strong predictive value regarding mortality in patients undergoing valve replacement for infective endocarditis. In addition, VIS showed superiority over conventional scoring systems for monitoring hemodynamics in the intensive care unit for postoperative IE patients.

### Limitations

This is a retrospective single-center study with the inherent limitation of such an analysis. The small number of patients is associated with a low power of statistical analyses. The VIS was calculated as the sum of the maximum dose of all inotropes or vasopressors administered during the study period. Consequently, the VIS is not a suitable method for assessing the individual impact of each drug on the outcome.

## Data Availability

The datasets generated and/or analyzed during the current study are not publicly available due to national data safety regulations and are available from the corresponding author upon reasonable request.

## Abbreviations

AUC: Area under the curve
BCNIE: Blood culture negative infective endocarditis
BSA: Body surface area
CI: Cardiac index
CKD: Chronic kidney disease
CO: Cardiac output
COPD: Chronic obstructive pulmonary disease
CPB: Cardio pulmonal bypass
DAPT: Dual antiplatelet therapy
EuroSCORE II: European System for Cardiac Operative Risk Evaluation II
FFP: Fresh frozen plasma
Hb: Hemoglobin
ICU: Intensive care unit
IDDM: Insulin dependent diabetes mellitus
IE: Infective endocarditis
MCS: Mechanical circulatory support
NOAC: Novel oral anticoagulants
PAD: Peripheral arterial disease
PCI: Percutaneous coronary intervention
POD: Postoperative day
PRBC: Packed red blood cells
PTCA: Percutaneous transluminal coronary angioplasty
ROC analysis: Receiver operating characteristic analysis
SaO_2_: Arterial oxygen saturation
SAPT: Single antiplatelet therapy
ScvO_2_: Central venous oxygen saturation
TAVR: Trans-aortic valve replacement
VIS: Vasoactive inotropic score
VISmax: Vasoactive inotropic score maximum
